# Hyperthyroidism with Biventricular Heart Failure and Cirrhotic Transformation of the Liver

**DOI:** 10.1155/2018/3861340

**Published:** 2018-12-09

**Authors:** Rashmi Dhital, Shivani Vyas, Priyadarshani Sharma, Theresa Lynn, Oreoluwa Oladiran, Sijan Basnet

**Affiliations:** Reading Hospital, Tower Health System, West Reading, PA, USA

## Abstract

Cardiovascular symptoms remain the most common presenting features and leading causes of death in hyperthyroidism. We report a young female with reported thyroid disease and medication noncompliance presenting with atrial fibrillation, severe atrioventricular regurgitation, severely dilated right heart with reduced function, and moderate pulmonary hypertension (PH), which was further complicated by congestive liver injury with ascites and pancytopenia. Thyroid work-up revealed suppressed TSH, elevated free T4 and T3 along with elevated anti-thyroglobulin antibodies, thyroid peroxidase antibodies, and thyroid-stimulating immunoglobulin, suggesting Graves' thyrotoxicosis. Ultrasound of the abdomen was suggestive of liver cirrhosis and ascites, which was thought to be cardiac cirrhosis, after multiple negative work-ups for alternate causes of cirrhosis. Ascitic fluid analysis revealed portal hypertension as the cause. The patient was restarted on antithyroid medication with gradual improvement of thyroid function and in clinical and echocardiogram findings. In contrast to primary PH that carries a poor prognosis and has limited treatment options, PH due to Graves' disease carries a good prognosis with prior reports of resolution after appropriate treatment, emphasizing the importance of early recognition. Also, unlike cirrhosis caused by alcohol or viral hepatitis, the effect of cardiac cirrhosis on overall prognosis has not been clearly established.

## 1. Introduction

Cardiovascular symptoms remain the most common presenting features and leading causes of death in hyperthyroidism [1, 2]. Varied cardiac presentations include atrial fibrillation (AF), chamber enlargement (more often right-sided), congestive heart failure (CHF), valvular regurgitation (atrioventricular more than semilunar), and pulmonary hypertension (PH), most of which have been reported to reverse with correct treatment [2–6]. Here, we present a case of a severely dilated right heart with moderately reduced function, severe atrioventricular regurgitation, and moderate PH due to medication noncompliance in a young female with Graves' disease, which was further compounded by congestive liver injury and decompensated cirrhosis.

## 2. Case Report

**Figure 1:**
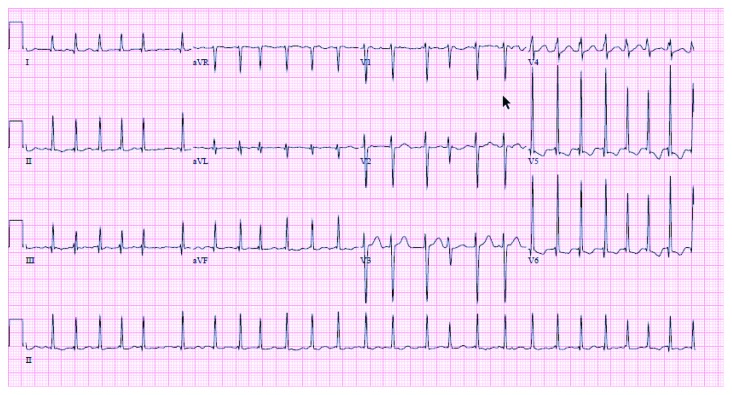
Atrial fibrillation with rapid ventricular rate (at presentation)—HR: 156 beats/min.

A 31-year-old female presented with abdominal distention, leg swelling, and dyspnea on exertion. She denied past medical history except for a thyroid condition for which she was on and off of medications. Examination revealed conjunctival pallor and scleral icterus. She was afebrile and normotensive but tachycardic with heart rate 160-190/minute. Cardiovascular exam revealed an irregular rhythm, systolic murmur at the lower left sternal border and cardiac apex, and an elevated JVP. Chest radiograph displayed cardiomegaly. EKG showed atrial fibrillation with rapid ventricular response (Figure 1). She received metoprolol with adequate rate control. Brain natriuretic peptide was over 4000. Urine drug screen was negative. Transthoracic echocardiogram (TTE) reported an ejection fraction (EF) of 43%, global hypokinesia, grade 2 diastolic dysfunction, anterior mitral valve prolapse (MVP), very severe mitral regurgitation (MR), severe tricuspid regurgitation (TR), severely dilated atria, and right ventricular enlargement with moderate PH.

Thyroid work-up revealed suppressed TSH at <0.005 (ref: 0.45 – 5.33) uIU/ml, elevated free T4 at 5.36 (ref: 0.58 – 1.64) ng/dl, free T3 of 28.31 (ref: 2.2-4.10) pg/ml along with elevated anti-thyroglobulin antibodies at 12 (ref: ≤4) IU/ml, thyroid peroxidase antibodies at 3841 (ref: ≤8) IU/ml, and thyroid-stimulating immunoglobulin >500% (≤122%), suggesting Graves' thyrotoxicosis. Thyroid ultrasound showed significantly enlarged, mildly heterogeneous lobes without discrete nodules. Methimazole was started with plan for subsequent radioactive iodine ablation.

Other notable labs included elevated alkaline phosphatase, bilirubin, and international normalized ratio with normal transaminases. Subsequent ultrasound of the abdomen showed moderate ascites with liver architecture suggestive of cirrhosis. Paracentesis removed 4.4 l of ascitic fluid with serum ascitic albumin gradient (SAAG) over 1.1, suggesting portal hypertension. She denied alcohol use and had no other risk factors for nonalcoholic liver disease including obesity (low normal body mass index of 18.4 kg/m^2^), systemic hypertension, dyslipidemia, or diabetes. Work-up was negative for immune causes of cirrhosis, including antinuclear antibody (ANA), ceruloplasmin, alpha-1-antitrypsin (AAT), anti-mitochondrial antibody (AMA), and anti-smooth muscle antibody (ASMA). Ferritin level was within range, and the hepatitis panel was negative.

The patient was discharged home on a beta-blocker and diuretics. Follow-up office visit showed improving thyroid functions TSH at <0.005 (ref: 0.45 – 5.33) uIU/ml but normal free T4 at 0.60 (ref: 0.58 – 1.64) ng/dl, and slightly elevated free T3 at 4.28 (ref: 2.20 – 4.10) pg/ml. EKG showed normal sinus rhythm and normal rate. Subsequent echocardiogram at 2 months showed normal left ventricular systolic function with EF of 61%, no regional wall motion abnormalities, normal diastolic function, moderate MR and TR, and top-normal right-sided pressures. Ultrasound of the abdomen at that time showed persistent appearance of cirrhosis with improved ascites.

## 3. Discussion

Several years of research has shown that hyperthyroidism has cellular, molecular, and hemodynamic effects on the cardiovascular system, with varied clinical presentations [2, 4, 5, 7]. Myocellular effects comprise of increase in myocellular contraction with subsequent increase in oxygen demand among cardiac myocytes [8, 9]. Hemodynamic effects include increased preload (by increased diastolic relaxation and renin-angiotensin-aldosterone activation) and decreased afterload (decreased systemic vascular resistance) [2, 10]. These myocellular and hemodynamic alterations can lead to tachyarrhythmias, thyrotoxic cardiomyopathy, decreased exercise tolerance, and rapid decompensation [2]. In their study of thyrotoxic heart disease patients, Wu et al noted 63% to have heart failure, 60% to have MR and TR, and 44% to have PH [5]. In uncontrolled hyperthyroidism, impaired collagen metabolism may lead to myxomatous leaflet and chordae degeneration, with subsequent MR [3, 4, 11, 12]. Another probable cause of valvulopathy is the hemodynamic changes in thyrotoxicosis, including increased venous return and dilatation of cardiac chambers and valve annulus [2, 13]. Serious cardiopulmonary symptoms from uncontrolled hyperthyroidism can be detrimental.

Potential explanations for PH include autoimmune or high cardiac-output-mediated endothelial damage in addition to elevated pulmonary vascular resistance due to decrease in pulmonary vasodilators and an increase in vasoconstrictors [9, 14]. Also, in hyperthyroidism, there is an accelerated metabolism of intrinsic and extrinsic pulmonary vasodilators (prostacyclin, nitric oxide, and acetylcholine) and an impaired metabolism of pulmonary vasoconstrictors (serotonin, thromboxane, and endothelin-1) [14, 15]. PH, if left untreated, can cause increased right ventricular afterload with right heart dilatation and failure, which is compounded by functional TR by annulus dilatation [16].

This right heart failure in long-standing hyperthyroidism can, in turn, cause passive liver congestion called congestive hepatopathy and clinical as well as histological changes of liver cirrhosis [13, 17]. Liver dysfunction may range from mild hyperbilirubinemia, coagulopathy, and hepatomegaly to ascites and liver cirrhosis. Khemichian and Fong have also described the association of Graves' disease with primary biliary cirrhosis or autoimmune hepatitis [13]. However, in our patient, the diagnosis of cardiac cirrhosis was thought more likely after multiple negative serological work-ups including ANA, AMA, and ASMA levels. Also, risk factors for other causes of cirrhosis were absent in our patient, including alcohol use and features typical for nonalcoholic fatty liver disease, including obesity, dyslipidemia, diabetes mellitus, or systemic hypertension.

Of the various manifestations of thyrotoxicosis, our patient presented with atrial fibrillation and tachycardia-induced cardiomyopathy, severe valvular disease, and PH, which was further complicated by congestive liver injury as evident by abdominal ascites with a SAAG >1.1. In contrast to primary PH that carries a poor prognosis and has limited treatment options, PH due to Graves' disease carries a good prognosis, with prior reports of resolution after appropriate treatment [3, 6, 11, 14], emphasizing the importance of early recognition. Also, it is very rare for hyperthyroidism-driven heart failure to lead to cirrhosis, and cirrhosis has been reported mostly in chronic right heart failure [18]. Unlike cirrhosis caused by alcohol or viral hepatitis, the effect of cardiac cirrhosis on overall prognosis has not been clearly established [18, 19]. By this case, we want to highlight the importance of considering hyperthyroidism early on in the differential for unexplained valvular regurgitation and heart failure and also considering cardiac cause as a differential for liver cirrhosis.
